# Cultivation and molecular profiling reveal ammonia-oxidizing archaea as skin commensals

**DOI:** 10.1093/ismejo/wrag078

**Published:** 2026-04-11

**Authors:** Alexander Mahnert, Maximilian Dreer, Ülkü Perier, Michael Melcher, Stefanie Duller, Adina Lehnen, Theodora Goessler, Daniela Brunner, Thomas Graier, Peter Wolf, Rafael I Ponce-Toledo, Logan H Hodgskiss, Melina Kerou, Christine Moissl-Eichinger, Christa Schleper

**Affiliations:** D&R Institute of Hygiene, Microbiology, and Environmental Medicine, Medical University of Graz, Neue Stiftingtalstrasse 6, 8010 Graz, Austria; Department of Functional and Evolutionary Ecology, Archaea Biology and Ecogenomics Unit, University of Vienna, Djerassiplatz 1, 1030 Vienna, Austria; Department of Functional and Evolutionary Ecology, Archaea Biology and Ecogenomics Unit, University of Vienna, Djerassiplatz 1, 1030 Vienna, Austria; Department of Functional and Evolutionary Ecology, Archaea Biology and Ecogenomics Unit, University of Vienna, Djerassiplatz 1, 1030 Vienna, Austria; D&R Institute of Hygiene, Microbiology, and Environmental Medicine, Medical University of Graz, Neue Stiftingtalstrasse 6, 8010 Graz, Austria; D&R Institute of Hygiene, Microbiology, and Environmental Medicine, Medical University of Graz, Neue Stiftingtalstrasse 6, 8010 Graz, Austria; D&R Institute of Hygiene, Microbiology, and Environmental Medicine, Medical University of Graz, Neue Stiftingtalstrasse 6, 8010 Graz, Austria; D&R Institute of Hygiene, Microbiology, and Environmental Medicine, Medical University of Graz, Neue Stiftingtalstrasse 6, 8010 Graz, Austria; Department of Dermatology and Venereology, Medical University of Graz, Neue Stiftingtalstrasse 6, 8010 Graz, Austria; Department of Dermatology and Venereology, Medical University of Graz, Neue Stiftingtalstrasse 6, 8010 Graz, Austria; Department of Functional and Evolutionary Ecology, Archaea Biology and Ecogenomics Unit, University of Vienna, Djerassiplatz 1, 1030 Vienna, Austria; Department of Computational, Quantitative and Synthetic Biology (CQSB), Sorbonne Université, CNRS, IBPS, UMR7238, Paris 75005, France; Department of Functional and Evolutionary Ecology, Archaea Biology and Ecogenomics Unit, University of Vienna, Djerassiplatz 1, 1030 Vienna, Austria; Department of Functional and Evolutionary Ecology, Archaea Biology and Ecogenomics Unit, University of Vienna, Djerassiplatz 1, 1030 Vienna, Austria; D&R Institute of Hygiene, Microbiology, and Environmental Medicine, Medical University of Graz, Neue Stiftingtalstrasse 6, 8010 Graz, Austria; BioTechMed Graz, Mozartgasse 12/II, 8010 Graz, Austria; Department of Functional and Evolutionary Ecology, Archaea Biology and Ecogenomics Unit, University of Vienna, Djerassiplatz 1, 1030 Vienna, Austria

**Keywords:** archaea, ammonia oxidation, human skin microbiome, commensalism, arrestin, host–microbe interaction, longitudinal microbiome study, comparative genomics, marker gene analysis

## Abstract

Ammonia-oxidizing archaea (AOA) have repeatedly been detected with molecular methods on human skin, yet their persistence, physiological traits, and adaptations remain poorly understood. This is mostly owed to a lack of cultured representatives of AOA taxa from healthy human skin. Using a customized enrichment scheme, we cultivated two autotrophic strains, *Candidatus* Nitrosocosmicus epidermidis and *Ca.* Nitrosocosmicus unguis, from human skin samples. Genomic analyses revealed specific adaptations for skin colonization, including genomic islands, and expanded gene families linked to interactions with host proteins, and signaling pathways, distinguishing these AOA from their soil-dwelling relatives. Profiling of >700 samples from 8 body sites in cross-sectional and longitudinal cohorts consistently validated the detection of *Ca. *Nitrosocosmicus** species with up to 100% prevalence in a longitudinal cohort, particularly in sebaceous areas. Co-occurrence patterns with specific bacterial taxa reinforce their role as stable components of the skin microbiome. Our results establish *Ca. *Nitrosocosmicus** species as common skin commensals that are evolutionarily capable of transitioning from soil to human skin. They likely play a critical role in the skin ecosystem by recovering nitrogen from the sebum through utilization of urea and ammonia. This sheds new light on the role of archaeal species in maintaining the nitrogen balance in the human skin microbiome, which might be of importance in maintaining healthy skin.

## Introduction

The human body is an ecosystem populated by spatially organized microbial communities composed of bacteria, archaea, viruses/phages, and small eukaryotes, with diverse metabolic activities and interactions: the microbiome [1]. In recent years, numerous studies have led to a better understanding of the mechanisms involved in the complex interplay between the host and its commensals and have revealed the fundamental role of the human microbiome in health and disease [2–4]. However, most studies on human microbial communities have focused exclusively on the bacterial microbiome, whereas other integral components, such as eukaryotes/fungi, archaea, and viruses, have remained largely overlooked [5–9]. A systematic metagenomic study recently revealed the extent of diversity and abundance of archaea in the human gut microbiome [10], where the increased presence of methanogenic archaea affects short-chain fatty acid production and overall host physiology [11].

The skin microbiome is similarly complex and harbors various bacterial species, including *Staphylococcus*, *Streptococcus*, *Corynebacterium*, and *Propionibacterium* (now *Cutibacterium*), and fungi such as *Malassezia* [12, 13]. The composition and diversity of the microbiome varies across different body sites (e.g. oily vs. dry skin) and between individuals [14]. As in the gut, microorganisms play essential roles in maintaining skin health as they provide protection against pathogens by competing for nutrients and space, producing antimicrobial compounds, and modulating the skin’s immune response [15, 16].

In addition to bacteria, human skin is also colonized by archaea [17, 18], in particular by representatives of the ammonia-oxidizing archaea (AOA), formerly *Thaumarchaeota*, now class *Nitrososphaeria* and phylum *Thermoproteota* [19–21]. Although rather rare in abundance, archaeal signals were found in human subjects analyzed by 16S rRNA gene sequencing and detection of archaeal *amoA*, the gene for subunit A of the key enzyme ammonia monooxygenase used for ammonia oxidation [17]. AOA signatures were detected on all human subjects and were closely related to those found earlier in cleanrooms and intensive care units [22]. Their presence was confirmed by fluorescence *in situ* hybridization [17], 454 pyro-sequencing [18], and infrared hyperspectral imaging [18]. Although the physiological relevance of AOA to the skin is still subject to speculation, its potential role is intriguing considering that urea and ammonia are components of human sweat and are the main energy sources of ammonia oxidizers. Also, nitric oxide (NO), an important signaling molecule involved in diverse processes in humans, is a byproduct of the ammonia oxidation process [23, 24].

Detection of archaea in human microbiomes is generally hampered by numerous methodological obstacles, such as challenging cultivation [25–27] and molecular detection issues (summarized in [28]), because of the bacteria-centric orientation in sampling, cell lysis, DNA extraction protocols, polymerase chain reaction (PCR) primers, and classification databases. Consequently, archaeal signals often remain close to or below the detection limit, making it difficult to understand the role of archaea on human skin [29]. Such obstacles and the lack of cultivated strains of skin AOA have left many questions unanswered, with respect to (i) abundance, (ii) longitudinal stability, (iii) association with human health, (iv) physiology and metabolism, (v) interaction with the bacterial microbiome, and (vi) specific adaptations to the human skin.

In this manuscript, we present the results of two complementary but independent approaches designed to investigate the persistence and characteristics of AOA on human skin: extensive cultivation efforts and molecular analyses targeting AOA in both healthy and diseased individuals. Our efforts yielded stable, highly enriched cultures of strains affiliated with the genus *Candidatus* Nitrosocosmicus from human skin. Their genomes reveal features that could facilitate colonization of the skin environment, but also highlight the genomic repertoire shared among all members of the genus *Ca.* Nitrosocosmicus, an environmentally widespread group of AOA. Molecular profiling further validated *Ca.* Nitrosocosmicus signatures across cohorts of healthy volunteers and patients, identifying correlations with physical parameters and bacterial co-occurrence patterns. Together, our findings suggest that specific *Ca.* Nitrosocosmicus species or clades have evolved distinct traits potentially enabling their transition from soil to specialized niches on human skin, supported by genomic adaptations, and their stable integration into the skin microbiome.

## Materials and methods

### Initial cultivation

Prior to enrichment setups, subjects not working in an experimental laboratory were pre-screened for the presence of *Nitrososphaerales* by targeted 16S rRNA gene amplification. We used three different primer pairs, two of which targeted 16S rRNA genes (Cren 771F/957R [30] and Arch 109F/1492R [31, 32]) and one targeting the *amoA* gene (19F/629R, [33, 34]). In dilution tests with template DNA primers, Arch 109F/1492R turned out to be the most sensitive and were thus used to screen individuals for the presence of AOA. All tested individuals (*n* = 41) were positive for AOA. In a first round of cultivation attempts, diverse swabs and brushes were tested to identify tools that are compatible with AOA growth. Sterile polyurethane single sponge swabs (BD BBL CultureSwab EZ; Becton Dickinson, USA) were identified as most suitable for enrichments because they promoted the growth of *Nitrososphaera viennensis* in pre-tests, whereas several other materials turned out to be inhibitory for growth.

For enrichment cultures, sterile swabs pre-moistened with sterile freshwater medium (FWM [35, 36]) were used to sequentially rub the face, neck, upper body, back, arms, and legs to obtain as much biomass as possible. All following procedures were performed under sterile conditions (laminar flow). The polyurethane swab was removed from the plastic shaft and directly added to FWM (enrichments T1S, X2B, and Z3A). For enrichment of R2S, free edges of a subjects’ fingernails (two to three clippings each obtained with a sterilized scissor) were directly added to FWM (enrichment R). Earlier studies had shown the suitability to use fingernail microbiomes to assess the skin microbiome due to scratching [37].

All initial enrichments were set up in sterile 30-ml polystyrene screw-cap tubes (Greiner, Austria) containing 20 ml FWM supplemented with 0.5 mM NH_4_Cl, 20 μl non-chelated trace element solution, 7.5 μM FeNaEDTA, 20 μl vitamin solution as well as 25 μg/ml carbenicillin, and 25 μg/ml kanamycin (for detailed medium composition, see table 1 in [38, 39]).

Variations between initial enrichments included the carbon source (2 mM Na_2_CO_3_ or NaHCO_3_), pH (7 or 8.5), temperature (28°C or 32°C), supplementation with organic substrates (0.5 mM acetate and 0.5 mM pyruvate or none), and filter sterilized spent growth medium of *Nitrososphaera viennensis* EN76 [36] and *Ca.* Nitrosocosmicus franklandianus C13 [40] (2.5% (v/v); Supplementary Table S1). Cultures were incubated under oxic conditions for up to several months without shaking and in the dark. First transfer of cultures was performed after 3 months, and *amoA* sequences and 16S rRNA gene sequences were determined for the initial enrichments that showed nitrite production as well as for their first transfers (see Supplementary Table S2). The sequences were identical in the original cultures and the corresponding transfer. For T1S enrichments, 6 different donors were sampled and 12 cultures (2 different growth conditions varying in organic acid addition) were set up. Out of 12 enrichments, one culture (and its subculture) was positive for ammonia oxidation and after further passages yielded T1S. In the case of R2S, one culture out of 10 setups (2 donor individuals) was positive. For the enrichments of X2B and Z3A, three individuals were sampled, and six initial setups yielded two positive cultures after 2 and 2.5 months, respectively. Sequences of 16S rRNA and *amoA* genes showed only few point mutations to the sequences of *Ca.* N. epidermidis.

Positive enrichments were routinely transferred (transfer volume 20% v/v) upon reaching nitrite concentrations of ~450 μM. After transferring subcultures R2S and T1S into a medium containing 10 μg/ml chloramphenicol, 10 μg/ml novobiocin, and 10 μg/ml streptomycin twice, no bacterial contaminants were detected, leaving the cultures with yeast contaminants. Both bacteria and fungi signatures were found in X2B and Z3A.

### Routine culture maintenance

Enrichments were routinely cultivated in sterile 30-ml polystyrene screw-cap tubes containing 18 ml FWM supplemented with 0.6 mM NH_4_Cl, 2 mM Na_2_CO_3_ (R) or NaHCO_3_ (T1S, X2B, or Z3A), 20 μl non-chelated trace element solution without molybdenum (Na_2_MoO_4_·2H_2_O), 7.5 μM FeNaEDTA, 20 μl vitamin solution as well as 25 μg/ml carbenicillin and kanamycin and were transferred every 2 to 4 weeks upon reaching nitrite concentrations of ~450 μM. The inoculation volumes were decreased to 10% (v/v).

The medium of enrichment R2S additionally contained 10 mM MOPS ((3-(*N*-morpholino) propanesulfonic acid), titrated to pH 7 with HCl) and had a final pH of 7. Ammonium and nitrite concentrations were measured colorimetrically by the indophenol method and Griess reagent, respectively, as described previously [38, 39]. Aliquots used for ammonium measurements were frozen at −20°C and measured jointly at a later time point. Cultures were incubated aerobically at 28°C without shaking in the dark. Solutions were autoclaved (FWM, NH_4_Cl) or filter sterilized with a 0.2-μm filter (trace element solution, vitamin solution, FeNaEDTA, antibiotics) prior to use (Supplementary Fig. S1: Enrichment Scheme).

### Microscopy

Cultures were 10× concentrated before being imaged with phase-contrast light microscopy using 60× and 100× oil immersion objectives. F_420_ autofluorescence was imaged using an F420 filter cube. For scanning electron microscopy, 6 ml of each enrichment at late exponential phase were concentrated by centrifugation (21 000×*g*, 4°C, 30 min). Pellets were fixed in 2.5% glutaraldehyde for 2 h, washed three times with 1× Phosphate-buffered saline (PBS), and resuspended in 100 μl 1× PBS. Then, 50 μl of the fixed cell suspension was spotted onto 0.01% poly-L-lysine–coated glass slides (5 mm) and sedimented for 2 h before being dehydrated in an ethanol series (30%–100%, 5 min each). The dehydrated cells were critical point dried in liquid carbon dioxide and subsequently coated with gold in a sputter coater. Samples were imaged in a JEOL IT 300 scanning electron microscope at 20 kV (CIUS imaging facility, University of Vienna).

### DNA extraction of cultures

High molecular weight (HMW) DNA of enrichment cultures was extracted by a combination of chemical lysis based on a modified lysis buffer for *Sulfolobus acidocaldarius* [41] and freeze-thawing, followed by a phenol chloroform extraction.

Between 0.3 and 0.5 l of each skin enrichment culture was concentrated by filtration (0.22 μm MF-Millipore mixed cellulose ester membrane filter), washed off the filter with FWM, and pelleted by centrifugation (21 000×*g*, 4°C, 30 min). Pellets were resuspended in a modified TENST buffer (20 mM Tris pH 8, 1 mM EDTA, 100 mM NaCl, 5% *N*-lauroylsarcosine, 0.1% Triton X-100) and incubated at 50°C for 1–2 h. Samples were subsequently subjected to three rounds of freeze-thawing (5 min at −70°C followed by 5 min at 70°C). Proteinase K was added to a final concentration of 100 μg/ml, and samples were incubated at 65°C for 1–2 h. After lysis, HMW DNA was extracted by a standard phenol chloroform extraction (2× phenol/chloroform/isoamylalcohol 25:24:1, 2× chloroform/isoamylalcohol 24:1) with low-speed centrifugation (4500×*g*, 4°C, 3 min) between steps. DNA was precipitated overnight at 4°C by adding 1 μl glycogen as a carrier and 2× volume of a 30% PEG6000 solution (v/v) with 1.6 M NaCl. The precipitated DNA was washed twice with 4°C cold 70% EtOH, air-dried at room temperature for 15–30 min, resuspended in 50–60°C nuclease-free water followed by an incubation at 55°C for up to 2 h. The extracted HMW DNA was quantified fluorometrically with a Qubit 2.0 Fluorometer (Invitrogen) and stored at −20°C.

### Quantitative PCR

Archaeal *amo*A genes were quantified in triplicate using primers *amoA*19F and *amoA*629R following previously established methods [26, 42] with some modifications: 20-μl reactions contained 10 μl Luna Universal qPCR Master Mix, 3 μl nuclease-free water, 0.5 μm of each primer, and 5 μl DNA template. DNA was amplified in a CFX Real Time PCR System (Bio-Rad) with the following cycling parameters: 95°C for 1 min, 40× cycles of 95°C 15 s denaturation, 60°C 30 s joint annealing-extension, and 60°C 30 s fluorescence measurement. Standard dilutions prepared from DNA of each enrichment respectively using primers *amoA*19F and *amoA*629R ranged between 10^2^ and 10^8^ copies μl^−1^. The efficiency of qPCR assays was between 86.1% and 93.0% with an *R*^2^ of ≥0.99.

### Sanger and NGS sequencing of marker genes and genomes

In order to confirm purity and identity of cultures, 16S rRNA genes were amplified by PCR with the archaea-specific primer A109f [31], the universal primer Univ1492R [43], the crenarchaea-specific primer pair 771F/957R [30], and the fungal ITS region–specific primer pair ITS1F and TW13 [44]. Amplification of the *amoA* genes was performed by PCR using the primers *amoA*19F [45] and *amoA*629R [33]. PCR products were purified using the Monarch PCR & DNA Cleanup Kit (New England Biolabs) before being sequenced.

### Genome sequencing, assembly, and comparative genomics

DNA of our enrichments was shotgun sequenced using the NovaSeq 6000 System (Illumina, paired-end, 150 bp) at Novogene. Reads were trimmed and Illumina adapters were removed using Trimmomatic (SLIDINGWINDOW:5:20 LEADING:5 TRAILING:5 MINLEN:50 HEADCROP:6) [46]. To obtain the full genomic sequences of the enriched archaeal communities from the selected cultures, Nanopore sequencing on MinION Mk1C (Oxford Nanopore Technologies plc., UK) was performed. For library preparation, extracted genomic DNA was repaired with the NEBNext Companion Module (New England Biolabs GmbH, GER) and then prepared for sequencing on a chemistry version 14 flow cell (R10.4.1, FLO-MIN114) following the ligation sequencing gDNA—Native Barcoding Kit 24 V14 (SQK-NBD114.24) and flow cell (FLO-MIN114) protocols.

Nanopore sequencing was performed according to the following configurations: run time of the MinION Mk1C device was set for 72 h with a pore scan frequency of 1.5 h, a minimum read length of 200 bp, high-accuracy base calling, and active channel selection, with reserved pores, read splitting, trimming barcodes, and mid-read barcode filtering enabled. The following software versions were used: MinKNOW v22.10.7, Bream v7.3.5, Configuration v5.3.8, Guppy v6.3.9, and MinKNOW Core v5.3.1. Resulting sequencing data were analyzed with the following specifications: First, simplex base calling was carried out on a GPU node at the Life Science Compute Cluster (LiSC) University of Vienna (Austria), using guppy-gpu v6.4.2 and the dna_r10.4.1_e8.2_260bps_sup.cfg model for superior base calling. Second, duplex base calling was achieved at a GPU Node at the LiSC University of Vienna (Austria), following the recommended tutorial on https://github.com/nanoporetech/duplex-tools using dorado v0.1.1 (https://github.com/nanoporetech/dorado) with the dna_r10.4.1_e8.2_260bps_fast@v4.0.0 fast model, followed by finding duplex pairs with duplextools v0.2.20-3.10 (https://github.com/nanoporetech/duplex-tools), and dorado stereo/duplex base calling with the dna_r10.4.1_e8.2_260bps_sup@v4.0.0 superior model. Merged simplex and duplex reads were then investigated by performing quality control using NanoPlot nanocomp v1.20.0 [47] followed by filtering with filtlong v0.2.1 (https://github.com/rrwick/Filtlong) (--min-length 200, --keep_percent 90, and --target_bases 20 million). Filtered reads were then assembled using flye v2.9.1 in --nano_hq and --meta mode with a target genome size of 3 Mbpm [48]. Reads were mapped with minimap2 v2.24 [49] and then polished with racon v1.5.0 (https://github.com/isovic/racon) using the following settings (-m -8 -x -6 -g -8 -w -500). Finally, consensus contigs were obtained with medaka v1.7.2 according to the r1041_e82_260bps_sup_g632 model (https://github.com/nanoporetech/medaka). Basic genome statistics were calculated with genometools by calling gt seqstat [50] and classified the genomes using gtdbtk v2.1.1 with the --full_tree option enabled [51]. Genome completeness and contamination were estimated with checkm2 v1.0.1 including the --allmodels option [52]. After genome comparison using drep v3.4.2 [53], genomes were finally annotated with eggnog-mapper v2.1.9 [54–59] including prodigal for gene identification. Marker genes were identified with Metaxa2 2.2 [60], and the remaining contaminants of enrichments were estimated with kraken2 2.1.2 and bracken 2.8 according to the PlusPFP database [61, 62] (https://benlangmead.github.io/aws-indexes/k2).

Gene prediction and annotation of the MAGs assembled in this study was performed automatically using the Microscope annotation platform from Genoscope [63], followed by extensive manual curation. Annotations were supplemented with arCOG assignments from the archaeal Clusters of Orthologous Genes database (2018 release) [64], Carbohydrate-active enzyme assignments (CAZymes) from dbCAN2 (v7.0) [65], and TCDB family assignments from the Transporter Classification Database [66], using scripts from [67].

Orthologous protein families from a dataset, including *Ca.* N. franklandianus C13, *Ca.* N. oleophilus MY3, *Ca.* N. hydrocola G61, *Ca. *N. agrestis** SS, and *Ca. *N. arcticus** Kfb, and the two newly sequenced Ca. Nitrosocosmicus isolates R2S and T1S were constructed using Orthofinder (v.2.5.4) with standard settings [68].

### Study cohorts for molecular-based analyses

Forty-seven healthy individuals (group A1: female = 11, male = 10, age = 20–40 years; group A2: f = 15, m = 11, age = 60–85 years) were recruited for an in-depth analysis of their skin microbiome, and a subgroup of high versus low skin archaeal carriers was selected for validation based on quantitative (*amoA* gene and 16S rRNA gene qPCR universal and archaea-specific primers; data types: Cquant1, Cquant2, and Cquant3; Supplementary Fig. 2) and qualitative (16S rRNA gene amplicons universal and archaea-specific primers; data types: Cqual1, Cqual2, and Cqual4; Supplementary Fig. 2) observations for longitudinal investigations (group B1 (from A1): f = 2, m = 4; group B2 (from A2): f = 5, m = 1) over the period of almost 2 years (six time points; data type: L). The sampling sites for A1/A2 were hand, outer arm, crook, armpit, forehead, part, décolleté, and back (*n* = 7), and for B1/B2: forehead, décolleté, arms, and back (*n* = 4). Supplementary Table S3 gives an overview on the demographic characteristics of all recruited subjects. Each subject completed a questionnaire with different categories, including height and weight, skin type, and detailed questions about their respective lifestyle about eating habits, smoking, alcohol consumption, skin care, medications, animal contact, and hobbies.

### Skin measurements and sampling

The forehead and the part, as well as both hands, forearms, crooks, and armpits, were swabbed with pre-moistened (0.9% (v/v) NaCl) swabs (one swab for each body site; BD BBL Culture Swab EZ; Becton Dickinson, USA). NaCl was baked at 260°C for 24 h before solving in PCR-grade water to remove DNA traces. The remaining body sites, including décolleté and back (~300 cm^2^), were sampled with a pre-moistened surface-sampling cellulose-sponge kit (VWR, 300-0229P). For the longitudinal sampling (cohort B1/B2, tp2–6), only the forehead was sampled with the swab. After skin sampling, samples were immediately frozen at −80°C until further processing. All skin sampling events were accomplished in a laboratory that never handled AOA, and controls from the environment, sampling procedure, and sample extraction were processed in parallel.

Measurements of the skin physiology were performed using the Cutometer MPA580 (Courage + Khazaka, Germany), using the Tewameter (to determine the barrier function by measuring the water loss; 30 s, 3 times), the pH-meter, the Sebumeter (to determine the sebum content, 30 s), and the Corneometer (to determine skin moisture). Each skin site was measured three times, and the mean of the measurements is reported in Supplementary Tables S4 and S5. Furthermore, the surface temperature of each region was determined using an infrared thermometer (Future Founder, China). The interpretation of skin parameters was done based on the recommended specifications by the manufacturer, which are provided in Supplementary Table S6.

### DNA extraction, controls, amplicon sequencing, and qPCR of skin samples

The DNA was extracted from swabs and sponges using the Purelink Microbiome DNA Purification Kit (rectal or environmental swab samples; Thermo Fisher Scientific, USA). For the extraction from sponges (liquid sample extract from décolleté and back), 1 ml of the sample was concentrated by centrifugation (20 817×*g*, 4°C, 10 min). The largest proportion of the supernatant was removed, and the remaining 70 μl was used for bead beating with 800 μl of the S1 lysis buffer. Material and process controls were included in each step to control for the DNA contamination of the used reagents. The DNA was stored at −20°C until further downstream analyses.

Various targets were assessed using amplicon-based analyses: (i) the archaeal 16S rRNA gene region (nested PCR approach as described in [17, 69]), the (ii) *amoA* targeted approach (described in [33, 36, 70]), and the (iii) “universal” approach, targeting the microbial 16S rRNA gene region [71]. All primer sequences and PCR protocols are provided in Supplementary Table S7.

Library construction and high-throughput sequencing were performed at the Core Facility Molecular Biology at the Center for Medical Research (Medical University of Graz, Austria). In a first step, the DNA concentrations of the generated amplicons were normalized using a SequalPrep normalization plate (Invitrogen), and subsequently each sample was indexed with a unique barcode sequence by 8 cycles of indexing PCR. These indexed samples were pooled and purified by gel cuts before the library was run on a MiSeq System (Illumina) and the Reagent Kit v3 with 602 cycles (2 × 301 cycles).

### Bioinformatics and data analysis

Resulting demultiplexed fastq reads were processed with QIIME2 [72] versions 2018.11 through 2023.2. After quality control, denoising of reads was achieved with DADA2 [73], and truncation of forward reads at a length of 230 bp and 120 bp for reverse reads was only used for the archaea-specific amplicon (519F-806R), whereas no truncation was set for all other amplicon constructs (universal: 515F-926R; *amoA*: 19F-629R). Resulting feature tables and representative sequences were deposited on our GitHub repository (https://github.com/CME-lab-research/SkinArchaeome). Potential contaminants were identified with decontam v.1.12 [74] using the prevalence mode with a threshold of 0.5 and were removed from downstream analysis. Representative sequences were classified by a pre-trained Naïve-Bayes classifier based on the curated and trimmed SILVA138 database [75] using RESCRIPt [76] and the feature classifier classify-sklearn plugin [77]. For phylogenetic metrics like UniFrac [78], a rooted phylogenetic tree was generated with FastTree2 [79] based on a masked MAFFT alignment. Data normalizations were achieved by scaling with ranked subsampling and the q2-srs QIIME2 plugin [80] at Cmin 50, 100, and 1000 followed by calculating core metrics for alpha and beta diversity. Feature differentials were determined with the QIIME2 plugins aldex2 [81], ANCOM [82], ANCOM-BC [82], and the R packages ANCOM2 (https://github.com/FrederickHuangLin/ANCOM-Code-Archive) and MaAsLin2 [83] including time as a fixed effect, and different subjects as a random effect where applicable, whereas feature loadings were determined with the QIIME2 plugin DEICODE [84]. Longitudinal analysis covering feature volatility over time as well as other numerical metadata categories like age, BMI, or skin physiology measurements including pH, skin fat, or skin wetness was realized with the q2-longitudinal plugin in QIIME2 [77]. The same plugin was used to calculate linear-mixed-effect models of Shannon diversity and weighted UniFrac dissimilarity along principal coordinate axis 1. Amplicons of the *amoA* marker gene were processed in a similar way without read truncations for DADA2 and read classifications by a pre-trained Naïve-Bayes classifier based on the *amoA* reference database [85, 86]. Connection of 16S rRNA to *amoA* gene information was achieved by following the tutorial at https://github.com/alex-bagnoud/Linking-16S-to-amoA-taxonomy/. Prediction of metadata categories was realized with the q2-sample-classifier QIIME2 plugin [77].

#### Custom python code for regressing all metadata categories:

To identify potential associations between taxa and recorded numerical, and categorical metadata, an ordinary least squares (OLS) regression fit method was used with python’s statsmodels smf formula (statsmodels.regression.linear_model.OLS - statsmodels 0.15.0 (+8)). Microbial counts of the different investigated cohorts were first CLR (center log ratio) transformed and filtered for <25% missing values. These values were then used as the response variable in dependence of each individual, their sex, age, BMI, body site, the overall microbial diversity indicated by the Shannon index, and where applicable the time of sampling. The model was then evaluated for each genus and recorded metadata according to their *Q* values (FDR-corrected *P* values) and estimated regression coefficients (python code available at GitHub: https://github.com/CME-lab-research/SkinArchaeome).

#### Network analysis

Network analyses were conducted to identify mutually exclusive and co-occurring clusters of archaea and bacteria in our merged (archaea-specific and universal) 16S rRNA gene amplicon dataset. After SRS normalization to Cmin = 100, TSS, and log transformations, the 40 most abundant taxa were represented as nodes within the network, where node size reflects taxa abundance and edges indicate only positive associations of Spearman correlations between pairs of taxa. Then, we used PCoA to ordinate the nodes in a two-dimensional plot, such that correlating nodes were close together and anti-correlating nodes were far apart. In addition, correlations of the network were further supported by the Sparse Co-Occurrence Network Investigation for Compositional data (SCNIC) package (https://github.com/lozuponelab/SCNIC) using the sparCC metric with default settings for filterings and the *R* value and bootstrap adjusted *P* values.

#### Phylogenomic tree reconstruction:

A genome database was created comprising the two AOA genomes presented in this study together with 141 MAGs and completely sequenced genomes (133 AOA and 8 non-AOA genomes) from IMG, DDBJ, or NCBI databases followed by protein prediction using Prodigal v2.6.3 [59]. A previously described workflow [87] based on the archaeal single-copy gene collection [88] was followed to identify phylogenetic markers (*e* value 10^−10^) to reconstruct the phylogenomic tree. A total of 36 ribosomal protein families encoded in at least 100 out of the 143 genomes present in our genome database were selected. Each protein family was aligned independently using MAFFT 7.520 [89] (L-INS-i algorithm) followed by the trimming of the alignments using BMGE [90] with default parameters. Trimmed protein families were concatenated using a custom python script, and the concatenated alignment was used to reconstruct a maximum likelihood (ML) phylogenomic tree in IQ-TREE v2.2.2.7 [91] under the LG + C20 + F + G model with 1000 ultrafast bootstrap replicates.

#### Phylogenetic analysis of 16S rRNA gene sequences

The identification of 16S rRNA gene sequences in our AOA genome database (see above) was performed using Barrnap v0.9 (https://github.com/tseemann/barrnap). The 16S rRNA gene sequence encoded in the MAG GCA_025930575.1 (NS-Epsilon) was used as a query in a BLASTN search against the NCBI database to improve the sequence representation of this sublineage with environmental sequences. Sequences were aligned with the SINA v1.7.2 aligner [92] using the SILVA database SSU Ref NR 99 release 138 [75] with default parameters followed by the alignment of the skin ASVs using MAFFT 7.520 [89] (--addfragments option, L-INS-i algorithm). The alignment was trimmed manually using AliView [93]. The ML phylogenetic tree was reconstructed using IQ-TREE v2.2.2.7 [91] with the best-fit model TIM2e + I + R3 chosen by ModelFinder (“-m MFP”) [94] according to Bayesian Information Criterion, SH-like approximate likelihood ratio test (“-alrt 1000”) [95], and 1000 ultrafast bootstrap replicates (“-bb 1000”).

#### qPCR-specific methods

A SYBR green–based quantitative real-time PCR (qPCR) approach targeting the archaeal *amoA* gene (19F-629R) and the 16S rRNA gene of archaea (A806F-A958R) and bacteria (Bac_331F-Bac_797R) was used to determine the abundance of AOA, all archaea, and all bacteria on selected body sites. Primers, reaction mixtures, and thermocycler programs are listed in Supplementary Table S7. All qPCRs followed the MIQE Guidelines. The qPCRs were performed with the Bio-Rad SsoAdvanced Universal SYBR Green Supermix. Each reaction contained 1 μl of DNA template and 9 μl of mastermix. The mastermix for the archaeal *amoA* approach consisted of 5 μl SsoAdvanced Universal SYBR Green Supermix (Bio-Rad), 1.25 μl of each primer (1.25 μM), and 1.5 μl of PCR-grade water. The mastermix for the archaeal and bacterial 16S rRNA gene approach consisted of 5 μl SsoAdvanced Universal SYBR Green Supermix (Bio-Rad), 0.3 μl of each primer (10 μM), and 3.4 μl of PCR-grade water. Amplifications were carried out in a Bio-Rad CFX96 Touch Real-Time PCR Detection System. The qPCR cycling conditions were as follows: initial denaturation at 95°C for 1.5 min, followed by 45 cycles of denaturation for 15 s at 95°C, annealing for 30 s at 60°C, elongation for 40 s at 72°C, a final denaturation for 15 s at 95°C, 5 s at 60°C, and a storage temperature of 8°C. A plasmid containing the *amoA* gene of *Nitrososphaera viennensis* with a starting concentration of 104.1 ng/μl (pscA_Ampkan_AmoA, clone 3) was used as a quantification standard in a 1:10 dilution series from 10^−2^ to 10^−10^. Product specificity was confirmed by melting curve analysis. All qPCR experiments were performed in triplicates, and the results were averaged for further calculations. Data from a previous experiment on the total archaeal community (based on qPCR of the 16S rRNA gene) were also included in the analyses. To account for the different area sizes of the sampled body sites, copy numbers were adjusted to 300 cm^2^ (*amoA* gene copies per 300 cm^2^; see Supplementary Table S8). The qPCR files were analyzed using the CFX Manager Software version 3.1 and processed with the R software, version 3.6.1, and Microsoft Excel.

## Results

### Cultivation of AOA from human skin

Different types of inocula were used to initiate enrichment cultures under oxic conditions in the presence of 0.5 mM ammonia as sole energy source. Pooled samples were used after swabbing the face, neck, upper body, back, arms, and legs of different individuals. After 2 to 9 months, amplification and sequencing of archaeal 16S rRNA and *amoA* gene fragments identified the presence of single AOA strains in initial enrichments. The usage of pooled samples of three different individuals resulted in three enrichment cultures: *Ca.* Nitrosocosmicus “T1S,” “X2B,” and “Z3A.” A fourth enrichment culture was obtained from cut fingernail edges as initial inoculum (“R2S”) (details of enrichment source are provided in Supplementary Table S2).

**Figure 1:**
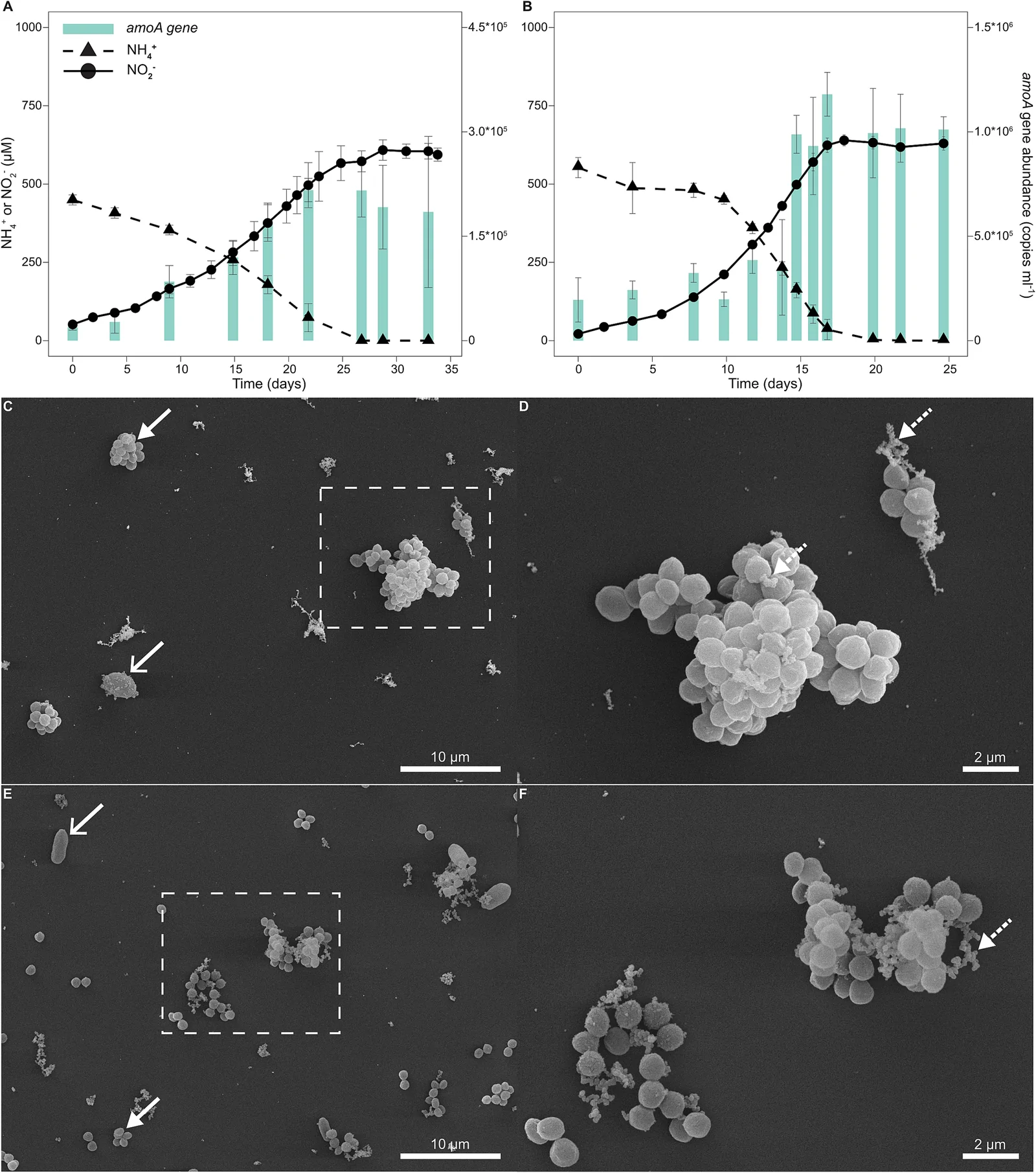
Growth and morphology characteristics of skin enrichments. R2S (A) and T1S (B) displaying near-stoichiometric conversion of ammonia (triangles, dashed line) to nitrite (circles, solid line) paralleled by cell growth, estimated by qPCR of archaeal *amoA* gene copies. Ammonia and nitrite measurements are averages of biological triplicates. Gene copies of *amoA* are averages of technical triplicates of each biological triplicate. Error bars depict the standard deviation of the mean. Scanning electron micrographs of skin enrichments R2S (C, D) and T1S (E, F) with white arrows indicating AOA cells based on the morphologies seen in Supplementary Fig. 3 and already published members of the *Ca.* Nitrosocosmicus [26, 40, 98, 99]. Dashed white arrows indicate putative extracellular polymeric substances. (D, F) Open white arrows indicate the remaining fungal contaminants.

The current study focuses on two of the enrichment cultures, “T1S” and “R2S,” isolated from skin swabs and fingernail clippings, respectively. These enrichments exhibited high enrichment of 88%–98% based on microscopy and molecular techniques (Supplementary Fig. 3) and stoichiometric conversion of ammonia to nitrite every 3–4 weeks (Fig. 1A, B) after repeated transfers over the course of more than a year in medium supplemented with 0.6 mM ammonium, 2 mM of either sodium carbonate (R2S) or sodium bicarbonate (T1S), and an antibiotic mix. No bacterial contaminants were detected via amplification of the bacterial 16S rRNA gene (Supplementary Fig. 4) or by light and scanning electron microscopy (Supplementary Fig. 3). Based on the sequenced internal transcribed spacer (ITS) region, the remaining contaminant in both enrichments was a single fungal species with 99% sequence identity to *Exophiala* sp. DAOM 216391, a genus of fungi frequently found in manmade environments and known to be an opportunistic pathogen of humans [96, 97]. Cell growth of the two strains R2S and T1S correlated with nitrite production as determined by quantification of *amoA* genes using qPCR, as has previously been shown for other AOA (Fig. 1A, B) [26, 36, 42]. The shortest generation times based on nitrite production were ~5 days at 28°C. After the oxidation of ~500 μM ammonium, enrichments R2S and T1S reached cell densities of 2.16 × 10^5^ (±1.68 × 10^4^) and 1.18 × 10^6^ (±1.05 × 10^5^) cells ml^−1^, respectively. This is similar to the cell densities of *Ca.* Nitrosocosmicus franklandianus C13 (3.8 × 10^6^ cells ml^−1^) [40] and *Ca.* Nitrosocosmicus oleophilus MY3 (1.6 × 10^6^ cells ml^−1^) [98] after oxidation of ~500 μM ammonium. For light microscopy, putative AOA were observed as cocci with diameters between 1 and 2 μm which frequently formed aggregates and were partly enclosed in putative extracellular polymeric substances (Supplementary Fig. 3). These morphological features are comparable with those of previously published members of the genus *Ca.* Nitrosocosmicus [26, 40, 98, 99] and were confirmed by scanning electron microscopy (Fig. 1C–F).

### Taxonomic placement and genomic features of the AOA representatives from human skin

Sequencing and assembly of the genomes of the two skin enrichments resulted in one circular contiguous sequence affiliated with AOA for each strain. The genome sizes, G + C content, and predicted protein-coding genes of the strains were well within the range of other representatives of the genus *Ca.* Nitrosocosmicus (Table 1). Like other representatives of this genus, both strains encoded three identical copies of the 16S and 23S rRNA gene operons. The average nucleotide identity (ANI) and average amino acid identity (AAI) values between strains R2S and T1S (87.4% ANI, 86.2% AAI), and between them and other *Ca.* Nitrosocosmicus genomes and proteomes, were well below the threshold of 95% ANI/AAI proposed for species delineation [100] (Supplementary Tables S9 and S10). We therefore propose that they represent two distinct species, preliminarily named *Ca.* Nitrosocosmicus epidermidis strain T1S (e.pi.der’mi.dis. Gr. fem. n. epidermis, the outer skin; N.L. gen. n. epidermidis, of the epidermis) and *Ca.* Nitrosocosmicus unguis R2S (un’gu.is. L. gen. n. unguis, of a fingernail).

**Table 1:** Genomic and growth features of *Ca.* Nitrosocosmicus epidermidis and *Ca.* Nitrosocosmicus unguis compared to other cultivated representatives of the genus *Ca.* Nitrosocosmicus.

**Organism** ^**a**^	**Status**	**Growth temperature (°C)**	**pH**	**Genome size (Mb)**	**No. of contigs**	**Completeness** **(%)**	**Contamination** **(%)**	**DNA GC** **(%)**	**Protein-coding genes**	**16S/23S/5S rRNA gene copies**	**Reference**
Nitrosocosmicus unguis R2S	Enriched	28	7	3.21	1	97.27	0.61	33.77	3597	3/3/1	This study
Nitrosocosmicus epidermidis T1S	Enriched	28	7	3.07	1	99.94	2.20	33.55	3401	3/3/1	This study
*N.* oleophilus MY3	Pure	30	6.5–7	3.43	1	99.76	0.54	34.14	3722	3/3/1	Jung *et al.* 2016
*N*. franklandianus C13	Pure	40	7	2.84	1	99.22	0.15	34.07	3025	2/2/1	Lehtovirta-Morley *et al.* 2016
*N. arcticus* Kfb	Enriched	28	6	2.65^*^	23	98.67	0.19	34.00	2970	3/3/1	Alves *et al.* 2019
*N.* hydrocola G61	Enriched	33	8	2.99	1	99.76	0.48	33.94	3162	2/2/1	Sauder *et al.* 2017
*N. agrestis* SS	Enriched	37	6.5–7	3.22	43	96.10	2.91	33.42	3524	2/2/1	Liu *et al.* 2021

a All organisms have Candidatus status

**Figure 2:**
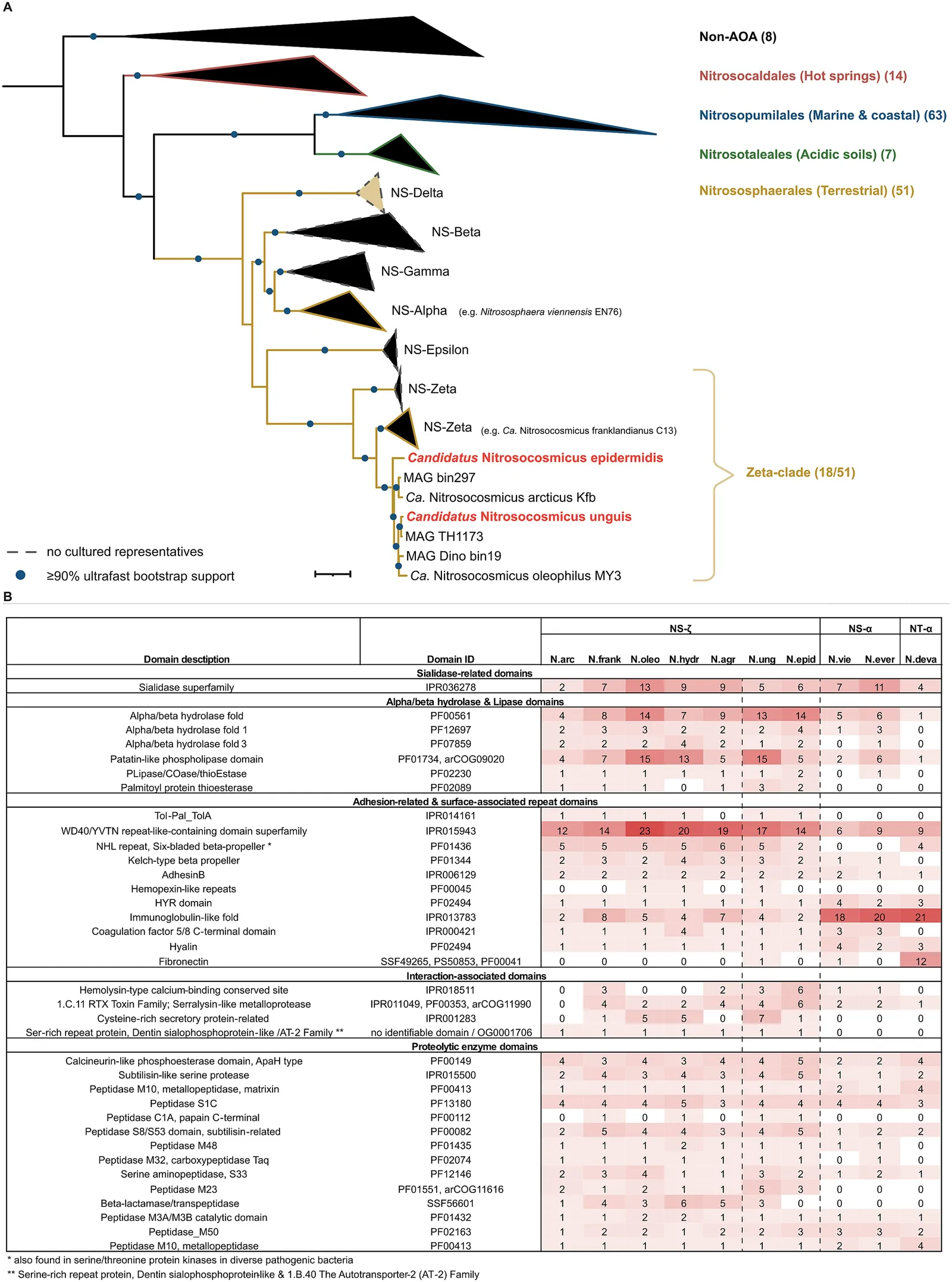
Comparative genomics of skin enrichments. (A) Maximum-likelihood phylogenomic tree of AOA and non-AOA reconstructed based on the concatenated alignment of 36 ribosomal proteins comprising a total of 4707 amino acid sites. Numbers in parentheses represent the number of complete genomes and MAGs in each lineage. NS = Nitrososphaerales. Tree scale = 0.1. (B) Heatmap displaying the detection frequency of specific functional domains discussed in the text in all analyzed *Ca.* Nitrosocosmicus genomes. Numbers refer to the number of occurrences of the respective domain in the genome, whereas not always corresponding to distinct CDS (i.e. multiple domains on one CDS). Domain IDs refer to Interpro, Pfam. TIGRfam, superfamily database entries.

A maximum-likelihood analysis based on 36 concatenated ribosomal protein marker genes present in one copy in at least 100 of the 143 genomes of a dataset comprising representative AOA and non-AOA of *Nitrososphaeria* confirmed the affiliation of the two strains with the NS-zeta lineage as previously defined [85]. Specifically, the two strains formed a highly supported cluster with *Ca. *N. arcticus** (obtained from Arctic soil), *Ca.* N. oleophilus (from coal tar–contaminated sediment), and other uncharacterized MAGs (Fig. 2 and Supplementary Tables S9–S11).

To investigate the specific genomic repertoire of *Ca.* N. unguis and *Ca.* N. epidermidis, we compiled a dataset consisting of the predicted proteomes of all available genomes from cultivated strains of the genus *Ca.* Nitrosocosmicus: *Ca.* N. franklandianus C13, *Ca.* N. oleophilus MY3, *Ca.* N. hydrocola G61, *Ca. N. agrestis* SS, and *Ca. N. arcticus* Kfb (we refrain from the “*Ca.*” prefix for better readability in the following). This protein dataset was clustered into 3301 orthologous protein families using Orthofinder (see Methods, Supplementary Data: Orthologs, and Supplementary Table S12). From these orthologous protein families, 1592 represented the core genome of the genus, comprising ~50% of the predicted CDS of the genomes (Supplementary Table S13). The NS-Zeta subclade containing N. epidermidis, *N. arcticus*, N. unguis, and N. oleophilus shared 60 protein families. N. unguis and N. epidermidis shared only 20 protein families to the exclusion of all others, whereas both of them shared more protein families with N. oleophilus (125 and 22, respectively) than any other Nitrosocosmicus genome. The shell (=strain-specific) proteome of each skin isolate consisted of 17 and 16 families for N. unguis and N. epidermidis, respectively. The content of arCOG functional categories did not differ among the Nitrosocosmicus genomes, indicating that presence and survival on human skin did not require a particular expansion in a functional category (Supplementary Fig. 5).

### Genomic island in *Ca.* N. epidermidis and protein families putatively involved in host interactions

The *Ca.* N. *epidermidis* genome contains genomic islands flanked by integrases, encoding proteins potentially involved in host interactions (Supplementary Data: Annotations). One such island (Nepid_2202–2261) includes a putative autotransporter-1 (AT-1) family protein (TCDB: (1.B.12), Nepid_2213) with extracellular domains of unknown function. These proteins typically use a C-terminal β-barrel to export N-terminal virulence factors [101]. In *Ca.* N. epidermidis, leucine-rich repeats on the N-terminal could facilitate protein–protein recognition [102]. The island also encodes a putative α-arrestin-like protein (Nepid_2245), containing arrestin N- and C-terminal domains (IPR011021 and IPR014752), which in eukaryotes bind to and regulate 7TM and kinase receptors (e.g. GPCRs) [103]. Though uncharacterized in prokaryotes, some pathogens exploit host β-arrestin pathways to traverse epithelial barriers [104]. Additionally, the island includes eight membrane-bound serine/threonine kinases and four putative serine-rich adhesins, known in streptococci for mediating tissue and extracellular matrix binding [105].

*Ca.* N. *unguis* encodes seven cysteine-rich secretory (CAP) family proteins (vs. one in *Ca.* N. *epidermidis* and 0–5 in other *Ca.* Nitrosocosmicus genomes, absent in NS-alpha and NT-alpha genomes, Fig. 2B; Supplementary Data), a group associated in eukaryotes with adhesion, signaling, and ion channel regulation, and potentially linked to pathogenesis or antimicrobial defense [106, 107].

Compared to environmental members of the genus, the skin-associated strains show an increased copy number (2–3 versus 1 in other *Ca.* Nitrosocosmicus and zero in other terrestrial AOA) of predicted extracellular/membrane attached putative triacylglycerol esterases/lipases (arCOG06923, PF02089) (Supplementary Data: Annotations, Fig. 2B). They are also among the *Ca.* Nitrosocosmicus genomes with the highest representation of alpha/beta hydrolase families, including patatin-like phospholipases, as well as adhesion-related and interaction-associated domains (Fig. 2B) [108]. Whereas the expression of lipases or esterases for degradation of host fatty acids in order to alter host immune response or utilize them for anabolic purposes is one of the most widespread mechanisms during bacterial colonization/infection of host epithelia [109, 110], no evidence for a complete β-oxidation pathway was found in the skin-associated strains or any other *Ca.* Nitrosocosmicus genome. This suggests that the lipase and hydrolase families are unlikely to function in fatty acid catabolism for energy generation. Nevertheless, microbial lipases can catalyze the hydrolysis of glyceride and ester substrates in lipid-rich surfaces, facilitating nutrient release and surface modification [109–113] (see also Supplementary Information).

### Archaeal ammonia oxidizers form a rare yet prevalent population on human skin

A complementary but independent approach was used to validate and understand the abundance, prevalence, distribution, and stability of archaeal and particularly *Ca.* Nitrosocosmicus representatives on human skin. To achieve this, 47 healthy individuals were independently recruited and eight defined body locations were sampled (cohorts A1 (age 20–40 years), A2 (60–80+ years), study overview: Supplementary Fig. 2 STORM chart, data types C). According to their microbial profiles (high AOA, low AOA, see Methods), 12 of these individuals were then selected and longitudinally sampled on four body sites for an entire year (cohort B; study overview: Supplementary Fig. 2, data types L). Samples were quantitatively (qPCR) analyzed using taxonomic (16S rRNA genes) and functional (*amoA* genes) markers, targeted toward the detection of archaea and archaeal ammonia oxidizers (AOA). As metagenomic sequencing failed due to the low biomass and high background in human DNA, diversity assessments of the skin archaeome were performed via amplicon sequencing to profile the bacterial, archaeal, and AOA-specific signatures. In total, 1770 amplicons (PCR amplifications (16S rRNA gene, *amoA*) per sample including controls) were processed for this validation (for details, see Materials and methods, Supplementary Fig. 2, and Supplementary Table S14).

**Figure 3:**
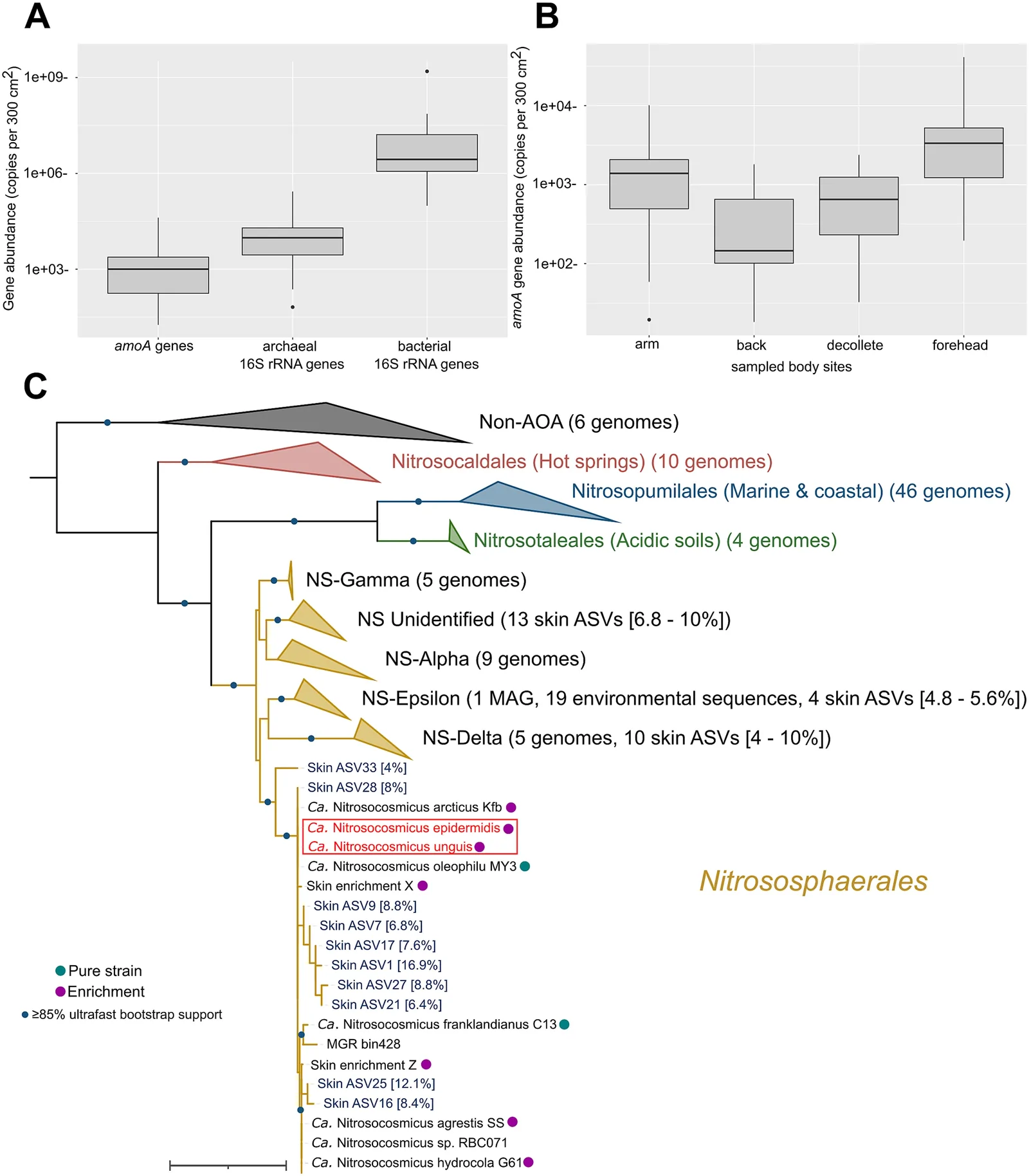
Quantitative and qualitative linkage between cultivation and molecular profiling. (A, B) Quantitative analysis (qPCR) of AOA and archaeal and bacterial 16S rRNA gene signatures on human skin (*n* = 284; data types: Lquant1, Lquant2, and Lquant3; Supplementary Fig. 2). The median of 16S rRNA gene copies across all sampling sites is shown; data are corrected for different 16S rRNA gene copy numbers in archaea (median: 1) and bacteria (6) (https://rrndb.umms.med.umich.edu/). Gene copies per 300 cm^2^ of AOA, archaea, and bacteria, shown on a logarithmic scaled *y*-axis. (B) Arm vs. back (*P* value 5.9 × 10^−4^), arm vs. décolleté (*P* value 7.2 × 10^−4^), arm vs. forehead (*P* value 2.1 × 10^−12^), back vs. forehead (*P* value 5.3 × 10^−13^), décolleté vs. forehead (*P* value 6.1 × 10^−13^). *AmoA* gene copies per 300 cm^2^, across sampling sites. (C) Maximum-likelihood phylogenetic tree of 16S rRNA gene sequences of AOA and non-AOA, including most prevalent and abundant ASVs of cohorts A and B. Blue sequences represent ASVs retrieved from amplicon sequencing of skin samples (cohorts A, B). Values in brackets show the percentage of prevalence of the ASV in our samples. Tree scale = 0.1.

Throughout the study (cohorts A, B), archaeal signatures were found at least once in all study participants, but were overall low, according to qPCR measurements (~0.15 ± 0.1% relative abundance, average across all body sites; data types Cquant1, Cquant2, Cquant3). AOA-specific qPCR signals accounted for 1.54 × 10^3^ gene copies per 300 cm^2^ (Fig. 3A). The highest amount of *amoA* gene copies were found in samples from the forehead (Fig. 3B); there, AOA signatures were found in samples from every tested person at each time point (prevalence: 100%; data types Lquant1, Lquant2, and Lquant3).

For amplicon sequencing, on average 6.9 ± 4.7% of all samples contained archaea when the universal PCR primer combination was applied (Supplementary Table S15, data types: Cqual1 and Lqual1; Supplementary Fig. 2), whereas an average of 70.0 ± 18.1% were positive with the archaea-targeted combination of 16S rRNA gene primers (Supplementary Table S8, data types: Cqual2 and Lqual2; Supplementary Fig. 2), emphasizing once more the necessity to use archaea-targeted methodology for optimized detection. Also, with this approach, all individuals were found to be positive for AOA signatures at least once throughout the study period (on average 86 ± 25%; 1.1 × 10^5^ ± 2.1 × 10^5^ reads of all samples).

Our independent approach with 16S rRNA gene sequencing detected most abundant AOA-associated ASVs within the *Ca.* Nitrosocosmicus genus which matched the identified genus from the cultivated enrichments (Fig. 3C). The most prevalent archaeal family within 326 samples (cohorts A and B) was Nitrososphaeraceae (74% positive samples) with *Ca.* Nitrosocosmicus being the most prominent genus in this family (24%) (Supplementary Table S16; data types: Cqual2 and Lqual2; Supplementary Fig. 2). The joint classification of 16S rRNA to the *amoA* marker gene (see Methods for details) resulted in a prevalence of 38% for the NS-Zeta clade across all samples (Supplementary Table S16; data types: Cqual2 and Lqual2; Supplementary Fig. 2).

### *Ca.* Nitrosocosmicus signatures show longitudinal coherence and unique biogeographic patterns across body sites

**Figure 4:**
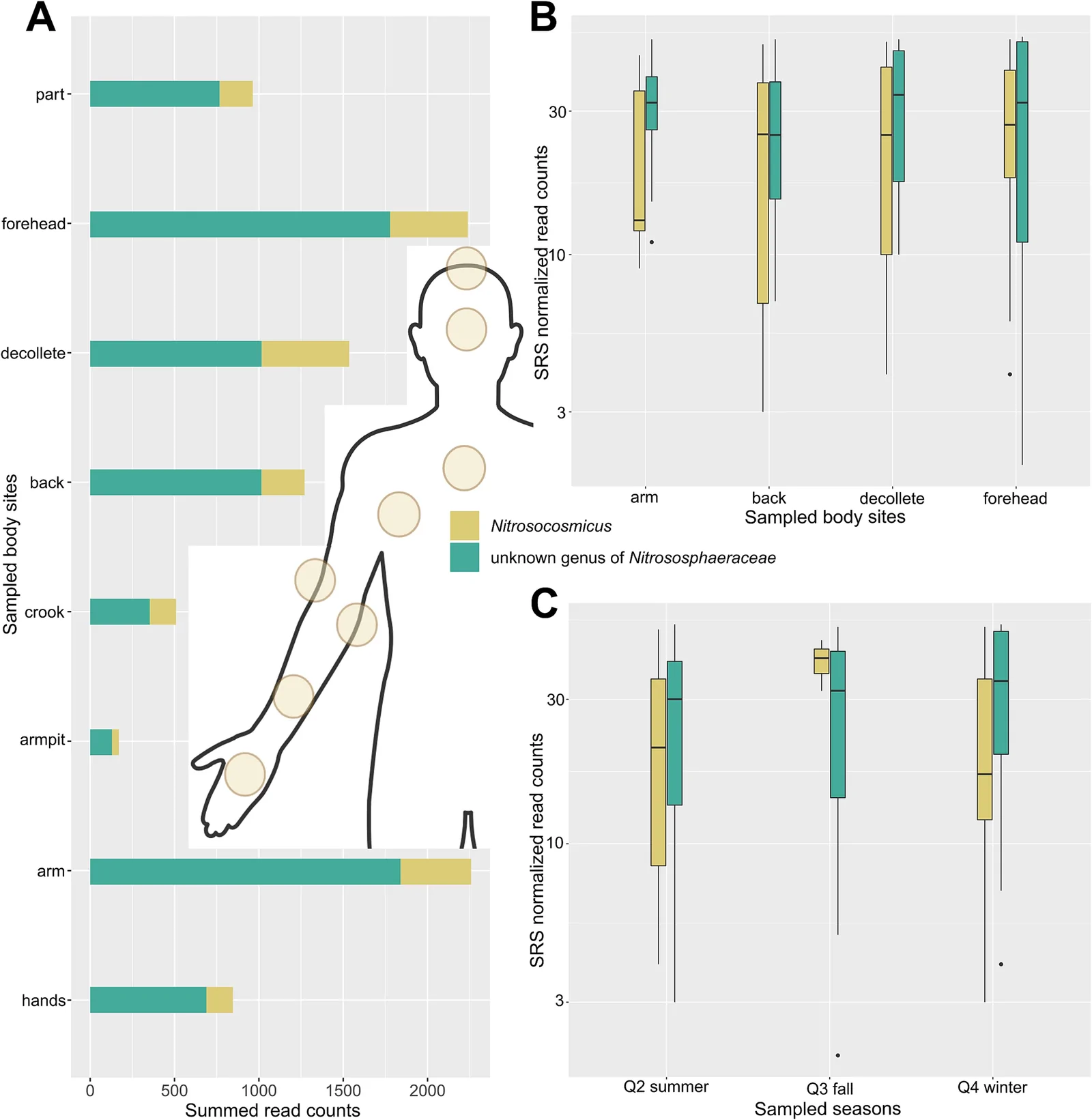
Biogeographic and longitudinal characteristics. (A) AOA prevalence across all samples from different body sites based on 16S rRNA gene amplicon sequences, shown by summed read counts (*n* = 326 samples; data types: Cqual2; Supplementary Fig. 2). The reads obtained were assigned to *Ca.* Nitrosocosmicus and other non-higher resolved genera belonging to the family Nitrososphaeraceae. Read counts and percentage of all sampled body sites for *Ca.* Nitrosocosmicus (part: 197, 9%; forehead: 461, 21%; décolleté: 519, 24%; back: 256, 12%; crook: 156, 7%; armpit: 41, 2%; arm: 418, 19%; hands: 157, 7%). Read counts and percentage of all sampled body sites for all AOA (part: 964, 10%; forehead: 2239, 23%; décolleté: 1535, 16%; back: 1271, 13%; crook: 509, 5%; armpit: 170, 2%; arm: 2258, 23%; hands: 846, 9%). The body silhouette was created using BioRender.com. (B) Boxplot showing the abundance of AOA (normalized read counts) per body site. (C) Boxplot showing AOA dynamics (normalized read counts) across the three covered seasons. Both (B) and (C) show read counts on a log-scaled *y*-axis and refer to the data of the longitudinal cohort (1 year, 6 time points, 3 seasons, 4 body sites, 283 samples; data type: Lqual2; Supplementary Fig. 2).

Across all eight body sites sampled (cohort A; data type: Cqual2; Supplementary Fig. 2), Nitrososphaeraceae and *Ca.* Nitrosocosmicus signatures were mostly detected on forehead, décolleté, back, and arms, indicating a skin physiology–dependent pattern of ammonia-oxidizing archaea in general (Fig. 4, 16S rRNA gene amplicons). High proportions of AOA from the head region could be confirmed quantitatively (qPCR), as highest copy numbers of the *amoA* gene (up to 4.1 × 10^4^ per 300 cm^2^) were retrieved from the head samples (part and forehead; Fig. 3B; data type: Lquant3; Supplementary Fig. 2).

*Ca.* Nitrosocosmicus signatures were stable longitudinally (1-year sampling period, Supplementary Information; data type: Lqual2; Supplementary Fig. 2), and participants who carried archaea at the initial time point consistently showed archaeal signatures above the detection limit throughout all subsequent time points (on average 78 ± 10%; 1.4 × 10^5^ ± 9.4 × 10^4^ reads of all samples). Seasons did not have a significant impact on the abundance of AOA (MaAsLin2, *Q* value = 0.95), indicating that contact with the outer environment (e.g. through contact to soil-born AOAs by gardening work (MaAsLin2, *Q* value = 0.49; data type: Lqual2; see Supplementary Fig. 2 STORM chart for more details)), did not influence the skin archaeome. We then tried to identify AOAs on genus level that followed a longitudinal pattern by regression analysis. Signatures assigned to *Ca.* Nitrosocosmicus (global mean = 0.17; global variance = 0.09; importance = 0.17) and other genera of the family *Nitrososphaeraceae* (global mean = 0.33; global variance = 0.15; importance = 0.12) achieved high importance in linear regression analysis over time supporting our assumption that AOA represent a coherent longitudinal trait on human skin (Supplementary Table S17; data type: Lqual2; Supplementary Fig. 2). Further information on bacterial ammonia oxidizers can be found in the Supplementary Information.

### AOA are an integral component of the healthy human skin microbiome

**Figure 5:**
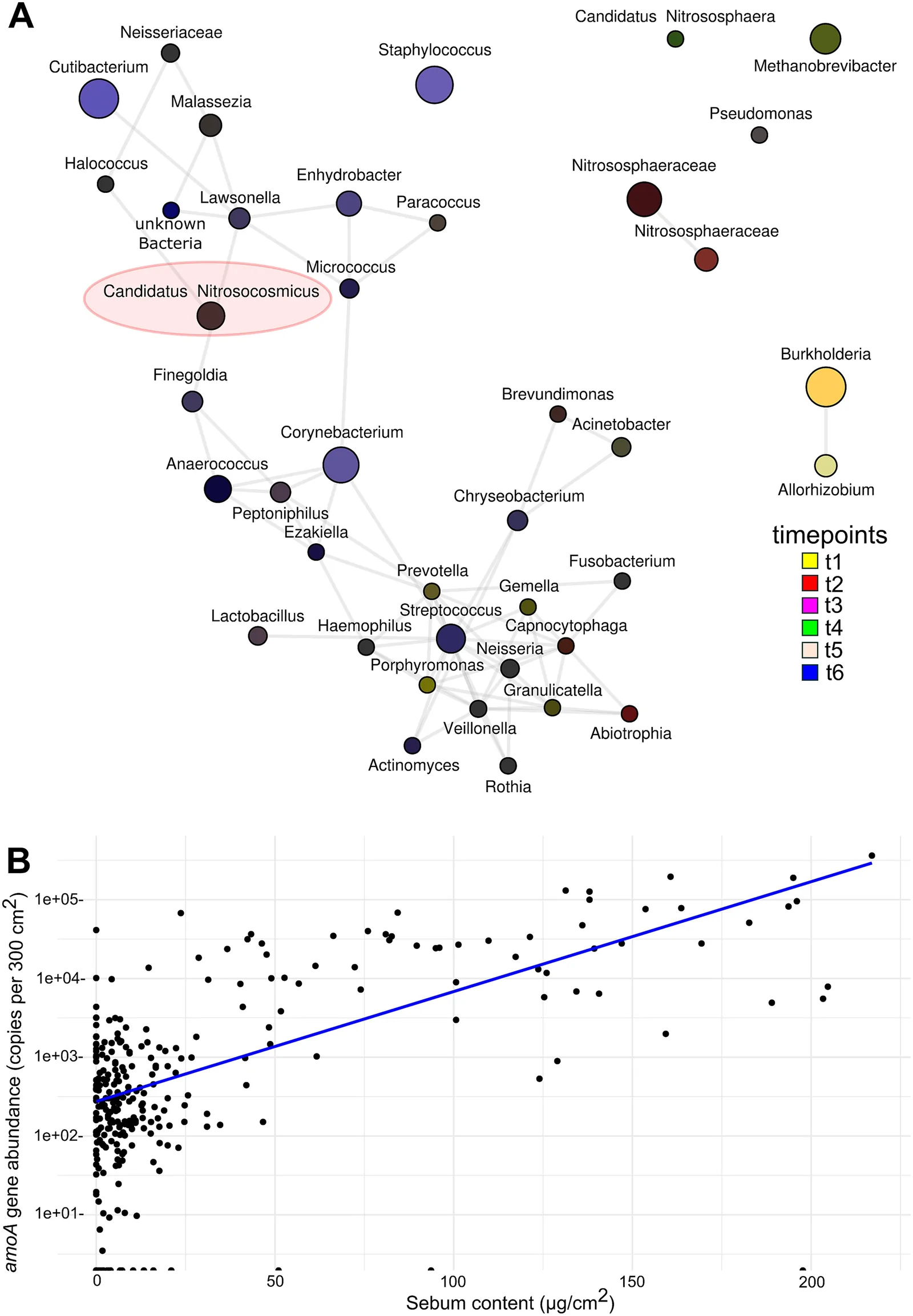
Metabolic niche within the human skin microbiome. (A) Microbial co-occurrence network (*n* = 283; data types: Lqual1 and Lqual2; Supplementary Fig. 2) between the 40 most abundant archaeal and bacterial taxa (SRS normalized Cmin = 100, TSS, and log transformed). Significant positive associations from Spearman correlations are highlighted by connecting edges. Taxa (nodes) were colored according to color mixtures of each sampling event (t1 yellow, t2 red, t3 pink, t4 green, t5 beige, t6 blue), resulting in dark gray colors for longitudinally consistent taxa in the dataset. The location of *Ca.* Nitrosocosmicus within the network is highlighted by a red ellipse. (B) Spearman correlation plot of AOA signatures and sebum content of the skin.

Network analyses (data types: Lqual1 and Lqual2; Supplementary Fig. 2) were performed to understand and validate the integration of AOA into the skin microbiome. These showed a significant positive correlation of *Ca.* Nitrosocosmicus with integral bacterial members of the human skin microbiome (in particular *Lawsonella* and *Finegoldia* SCNIC sparCC *P* adjust 0.26; see also initial *in silico* metabolic modeling in Supplementary Fig. 6). In contrast to other microbiome components, this interaction was relatively stable across all sampling time points (Fig. 5A; as visualized by the dark color of the node, see legend). A specific integration of *Ca.* Nitrosocosmicus into the skin microbiome network was underscored by the lack of positive correlations of other archaeal signatures that are not common on human skin (e.g. *Methanobrevibacter*).

OLS regression models were used to find associations between archaeal taxa and acquired metadata (skin physiology, demographics of the cohort, and their lifestyle as well as health parameters; data type: Cqual2; Supplementary Fig. 2). Considering the compositional nature of our amplicon datasets, we used CLR (center log ratio) transformed values and regressed them with all numerical and categorical metadata categories while taking care of several potential confounders (which partly had a significant impact on the microbial communities) like a subject’s sex, age, BMI, different sampled body sites, and the overall microbial diversity (Shannon Index). According to this analysis, signatures of *Nitrososphaeraceae* (*n* = 156) were significantly positively associated (*q* = 0.02) with qPCR-retrieved quantity for AOA (Supplementary Table S18). This finding supports and validates the congruence of amplicon-based qualitative and qPCR-based quantitative molecular analyses.

The abundance of *amoA* genes as detected by qPCR (tp1, *n* = 48) was not significantly influenced by sex (female vs. male), and age group (groups A1 (20–30 years) and A2 (>60 years)). However, the abundance of *amoA* signatures negatively correlated with pH (rho = −0.08, *P* value .17) and significantly negatively with increasing age (rho = −0.16, *P* value 7.7 × 10^−3^), trans-epidermal water loss (“Tewameter,” rho = 0.22, *P* value 4.8 × 10^−4^), and particularly sebum concentration (rho = 0.47, *P* value 1.0 × 10^−16^) according to Spearman rank correlations (data type: Cquant3; Supplementary Fig. 2). To assess the possibility whether AOA are involved in psoriatic disease, as hypothesized earlier [17], 20 subjects were recruited and *n* = 72 samples from lesioned and healthy skin samples were taken (cohort C, data type: P; Supplementary Fig. 2). However, according to differential abundance analysis based on ANCOM-BC (*P* adjust = .07) and MaAsLin2 (*Q* value = 0.54), samples from psoriatic skin did not reveal significantly higher or lower AOA counts (data type: Pqual2; Supplementary Fig. 2).

## Discussion

In this study, we demonstrate the consistent presence of AOA of the genus *Ca. *Nitrosocosmicus** on human skin, using two independent, but complementary, approaches: cultivation and molecular profiling. Collectively, our results suggest that *Ca.* Nitrosocosmicus representatives are an integral component of the skin microbiome. This conclusion is supported by (i) the repeated enrichment of *Ca.* Nitrosocosmicus strains from independent human skin samples, (ii) unique genomic features indicative of habitat-specific adaptation, (iii) their high prevalence and distinct biogeographic patterns in molecular analyses, and (iv) specific co-occurrence with established skin-associated bacterial taxa. Our findings not only highlight the ecological relevance of *Ca. *Nitrosocosmicus** on human skin but also raise important questions regarding their functional role. Both highly enriched AOA cultures exhibited a stoichiometric conversion of ammonia to nitrite that is typical of other isolated AOA species, and their genomes encode urease, enabling urea utilization. Given that ammonia and urea are the primary nitrogenous species excreted on human skin through sweat [114], these substrates likely represent a stable energy source for these organisms [115–117]. Over 50% of AOA species, including members of the *Nitrososphaerales* and the newly identified *Nitrosomirales* order, encode complete sets of urease genes (*ureABC*) and specialized transporters [116, 118, 119]. Through urea hydrolysis, these archaea convert urea into energy-yielding ammonia even when free ammonia is scarce [115, 116, 120]. This metabolic pathway is particularly advantageous on skin because it is not strictly pH dependent, allowing AOA to remain active within the skin’s naturally acidic environment [115]. These physiological traits, combined with high substrate affinity, enable AOA to persist within the fluctuating conditions of the human host [115].

Although the archaeal ammonia oxidation pathway has yet to be resolved, the intermediate nitric oxide (NO) has been observed to play a critical role for these organisms [24]. Because NO is one of the most important signaling molecules in mammalian physiology and is involved in a range of physiological as well as pathological processes on the human skin (reviewed in [121, 122]), AOA could contribute to the maintenance of NOx homeostasis on the human skin and thereby play a role in skin health or physiology. Also nitrite, the final product of nitrification, serves as a signaling molecule in a NO-independent manner [123, 124].

Both enriched strains were placed phylogenetically within the genus *Ca.* Nitrosocosmicus, a genus that we consistently detected and validated in cohorts A and B of our independent cross-sectional as well as longitudinal study. Although the relative abundance appears low in these human samples, it is not outside the range observed for AOA in other environments [26, 70].

To date, *Ca.* Nitrosocosmicus species have predominantly been isolated from soil environments and are known for their aggregate structures, propensity to grow in biofilms, and superior adhesion capacities [98, 99, 125]. Their potential to colonize healthy human skin is underscored by specific structural adaptations found in clades like *Nitrosocosmicus*, which lack a canonical S-layer and instead possess a thick glycocalyx covered in hair-like filaments [118]. This specialized outer layer enhances cell adhesion and supports the formation of biofilms, which serve as reservoirs that help the population resist physical displacement [115, 118]. These traits are supported by the specific encoding or expansion of adhesion proteins, acetamido sugar biosynthesis, and export proteins as well as protein families whose products can directly interact with and modulate other organisms and surfaces, such as proteolytic enzymes, alpha/beta hydrolases, phospholipases, and triacylglycerol esterases/lipases ([98, 99] and this study), which in the absence of a fatty acid catabolic pathway may contribute to colonization and remodeling of organic-rich surfaces, nutrient scavenging of minor lipid-derived intermediates, and modification of extracellular substrates [111–113, 126]. All of these features support the interaction of this genus with a variety of species in their respective environments and is further supported by recent evidence of *Ca.* Nitrosocosmicus associations and interactions with the plant rhizosphere [126]. The strains *Ca.* N. epidermidis and *Ca.* N. unguis share these genomics features, whereas also displaying unique attributes suggestive of putative functions that could facilitate colonization and persistence on an organic-rich environment such as the human skin.

Host interaction capabilities and attachment strategies are well established among skin-associated microorganisms [127], and our data indicate that *Ca. *N. epidermidis** exhibits similar potential. Its genome harbors a genomic island that is enriched in proteins that facilitate surface adhesion and interactions with various tissue types and proteins that could putatively interact with eukaryotic proteins, including an autotransporter [128] and arrestin-like protein [103, 104]. Similarly, *Ca.* N. unguis encodes multiple copies of cysteine-rich secretory protein (CAP) family proteins and beta propeller fold containing proteins that are both known for mediating interactions with the surrounding environment, including signaling and regulation of host proteins [106–108]. The distinct repertoire of each strain may reflect micro-habitat preferences on the heterogeneous skin landscape.

Currently, it remains difficult to unequivocally identify proteins that are specific to the skin environment, but this is not a *Ca.* Nitrosocosmicus–specific difficulty; many skin-associated microorganisms display ecological flexibility occupying both mammalian host–associated and environmental niches. Such dual lifestyles are well documented across microbial taxa. This observation suggests that colonization of the human skin in the case of *Ca.* Nitrosocosmicus does not require extensive lineage-specific innovation but may instead reflect ecological pre-adaptation of the genus to life on organic-rich surfaces, in the form of a surface-associated genomic repertoire that can facilitate colonization of diverse habitats, including soil particles, plant surfaces, and human skin (see also extended Discussion in Supplementary Information). This holds true for archaea in the case of the order *Methanomassiliicoccales*, which are able to thrive in both the gastrointestinal tract of mammals and diverse natural habitats [129–131], as well as for many opportunistic pathogens of bacteria including *Vibrio* sp. (aquatic environments [132]), *Stenotrophomonas maltophilia* (soil, plant environments [133]), or *Burkholderia pseudomallei* [134]. The constant interaction between host and environment can further confound the identification of host-related proteins without further in-depth analysis of individual strains.

The consistent presence of AOA on human skin within the longitudinal study supports the proposal of *Ca.* Nitrosocosmicus sp. being an emerging commensal skin microorganism. Their presence was found to be independent of age, sex, or time of year suggesting that they are a stable, and integral component of the microbial epidermal skin microbiome. The detected presence additionally showed correlations with skin parameters, in particular a positive correlation with sebum content. Sebum is an oily substance that contains many lipids with hydrophobic properties, and the correlation suggests that this AOA lineage can thrive in such an environment. This fits not only with the lipid interaction genes found within *Ca. *Nitrososcosmicus** but also with the placement of *Ca.* N. epidermidis, *Ca.* N. unguis, and several skin AOA ASVs next to the AOA species *Ca.* N. *oleophilus* which was isolated from coal tar–contaminated soil and exhibited a strong affinity for hydrophobic surfaces [98]. The positive correlations with both the genera *Lawsonella* and *Finegoldia* are also revealing as both have been associated with high sebum areas such as the forehead [135, 136]. These co-occurrence patterns may reflect ecological interdependencies or shared niche preferences.

The metabolic activity of AOA, particularly their role in nitrogen cycling, may be crucial in shaping the skin microbiome. Given the scarcity of ammonia-oxidizing bacteria on human skin, AOA likely fulfill this ecological function. Additionally, the capacity to produce crucial vitamins, such as B_12_ [137], via an autotrophic metabolism may also play an important role in the community structure of the skin microbiome as it does in other environments. Interactions of AOA with integral bacterial members of the human microbiome may also have a positive impact on the host that is so far unknown, as no association with psoriasis was found. Nevertheless, many aspects of skin health remain unexplored in the context of archaeal involvement. Based on our findings, we can conclude that *Ca.* Nitrosocosmicus is an integral and stable component of the microbial epidermal skin microbiome that is associated with relevant bacteria potentially via the exchange of ammonia, and is characteristic of healthy skin with higher sebum content, less moisture, and lower pH.

## Conclusions

This study marks the first AOA enrichments obtained from human skin and confirms the presence of the obtained genera, *Ca.* Nitrosocosmicus, on a wide range of individuals of varying sex and age. Comparative genomic and physiological observations suggest that colonization of the human skin by *Ca.* Nitrosocosmicus reflects ecological pre-adaptation of a surface-associated archaeal lineage, rather than recent host-driven genomic innovation. The enriched AOA are adapted for growth on organic-rich, hydrophobic surfaces such as human tissues, and both strains contain few unique protein families compared to other *Ca.* Nitrosocosmicus species that could facilitate either adhesion to or extracellular protein interaction with such a habitat. Further studies of skin archaea will be needed to reveal their evolutionary paths toward commensalism and to elucidate their roles in the skin microbiome and human health. The archaeal cultures obtained in this study are crucial prerequisites for advancing our knowledge in this area.

## Supplementary Material

AoHS_suppl_info_2026-03-01_ISMEJ_Rev_v6_wrag078

Archaea_on_human_skin_Supplementary_Tables_ISMEJ_v2_wrag078

Supplementary_Data_Annotations_revised_wrag078

Supplementary_Data_Orthologs_wrag078

## Data Availability

All raw sequencing data were submitted to NCBI’s Sequence Read Archive (SRA, https://www.ncbi.nlm.nih.gov/sra) and are accessible under the following projects: PRJNA1105887 (archaea-specific 16S rRNA gene amplicons of cohort A; data type: Cqual2), PRJNA1107153 (universal 16S rRNA gene amplicons of cohort A; data type: Cqual1), PRJNA1107462 (AOA-specific *amoA* gene amplicons of cohort A; data type: Cqual4), PRJNA1107502 (archaea-specific 16S rRNA gene amplicons of cohort B; data type: Lqual2), PRJNA1107512 (universal 16S rRNA gene amplicons of cohort B; data type: Lqual1), PRJNA1105887 (archaea-specific 16S rRNA gene amplicons of cohort C; data type: Pqual2), PRJNA1108073 (universal 16S rRNA gene amplicons of cohort C; data type: Pqual1), PRJNA1108908 (pooled shotgun metagenomics data of cohort A), PRJNA1108960 (Illumina sequencing data of enrichments), and PRJNA1109334 (Nanopore sequencing data of enrichments). Assemblies are publicly available under NCBI BioProjects PRJNA1103140 (*Ca.* Nitrosocosmicus epidermidis T1S) and PRJNA1103141 (*Ca.* Nitrosocosmicus unguis). Annotated assemblies are also available at the MicroScope Microbial Genome Annotation and Analysis Platform, developed by the Laboratory of Bioinformatics Analyses for Genomics and Metabolism, Genoscope, Evry, France.

## Code availability

The present study did not generate new code, and the mentioned tools used for the data analysis were applied with default parameters unless specified otherwise in Methods and our GitHub repo (https://github.com/CME-lab-research/SkinArchaeome).
